# Supplementary material for: Multimodal imaging and functional analysis of the chick NMDA retinal damage model
Source: PLoS One. 2021 Sep 7;16(9):e0257148. doi: 10.1371/journal.pone.0257148 (PMC8423281; doi:10.1371/journal.pone.0257148)
Supplement: S1 File — (ZIP) [file pone.0257148.s004.zip › SD-OCT/C200727/010/D28-Pwk5/C200727-010_OS_V_14X14_0_REGAVG0000133/Manual_PreDefineFiveByFiveGridRectangularRetina_1.pdf]

# Five By Five Grid Template Report

| | | | |
|---|---|---|---|
| **Patient ID** | C200727 | **Name** | See InVivoVue |
| **Exam Date** | See InVivoVue | **Exam Time** | See InVivoVue |
| **Eye** | OS | **Averaged** | N/A |
| **Depth (mm)** | 1.65 | **Depth Samples** | 1024 |
| **Pitch (um/pix)** | 35.000 x 35.000 x 1.608 | **Refractive Index** | See InVivoVue |
| **Scan Type** | Rectangular 14.000mm x 14.000mm x 1.647mm , 400 x 400 x 1024 | | |

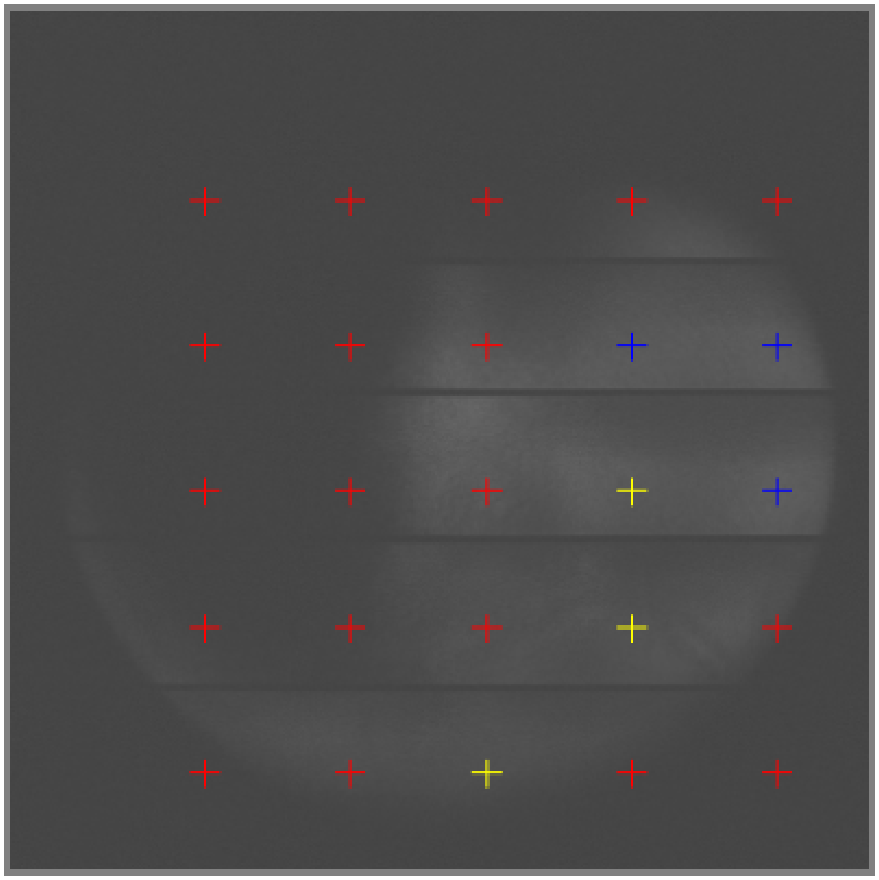

## Thickness Metrics

| Analysis | Average(mm) | Minimum(mm) | Maximum(mm) | STDEV(mm) |
|---|---|---|---|---|
| **IRNFL - RPE** | 0.2494 | 0.2453 | 0.2528 | 0.0038 |
| **IRNFL - ORNFL** | 0.0406 | 0.0343 | 0.0512 | 0.0092 |
| **ORNFL - OPL** | 0.1232 | 0.1173 | 0.1286 | 0.0056 |
| **OPL - ETPRS** | 0.0662 | 0.0649 | 0.0674 | 0.0012 |
